# Effect of Scaffold Geometrical Structure on Macrophage Polarization during Bone Regeneration Using Honeycomb Tricalcium Phosphate

**DOI:** 10.3390/ma17164108

**Published:** 2024-08-19

**Authors:** Kiyofumi Takabatake, Hidetsugu Tsujigiwa, Keisuke Nakano, Anqi Chang, Tianyan Piao, Yasunori Inada, Takuma Arashima, Ayumi Morimatsu, Ayumi Tanaka, Hotaka Kawai, Hitoshi Nagatsuka

**Affiliations:** 1 Department of Oral Pathology and Medicine, Graduate School of Medicine, Dentistry and Pharmaceutical Sciences, Okayama University, Okayama 700-8525, Japan; pir19btp@okayama-u.ac.jp (K.N.); pmff7em4@s.okayama-u.ac.jp (A.C.); pfd12zwy@s.okayama-u.ac.jp (T.P.); inayasu@s.okayama-u.ac.jp (Y.I.); de428001@s.okayama-u.ac.jp (T.A.); de428017@s.okayama-u.ac.jp (A.M.); phdo11rr@s.okayama-u.ac.jp (A.T.); de18018@s.okayama-u.ac.jp (H.K.); jin@okayama-u.ac.jp (H.N.); 2 Department of Life Science, Faculty of Science, Okayama University of Science, Okayama 700-0005, Japan; tsujigiwa@ous.ac.jp

**Keywords:** honeycomb TCP, bone formation, macrophages, polarization

## Abstract

The polarization balance of M1/M2 macrophages with different functions is important in osteogenesis and bone repair processes. In a previous study, we succeeded in developing honeycomb tricalcium phosphate (TCP), which is a cylindrical scaffold with a honeycomb arrangement of straight pores, and we demonstrated that TCP with 300 and 500 μm pore diameters (300TCP and 500TCP) induced bone formation within the pores. However, the details of the influence of macrophage polarization on bone formation using engineered biomaterials, especially with respect to the geometric structure of the artificial biomaterials, are unknown. In this study, we examined whether differences in bone tissue formation due to differences in TCP geometry were due to the polarity of the assembling macrophages. Immunohistochemistry for IBA-1, iNOS, and CD163 single staining was performed. The 300TCP showed a marked infiltration of iNOS-positive cells, which are thought to be M1 macrophages, during the osteogenesis process, while no involvement of CD163-positive cells, which are thought to be M2 macrophages, was observed in the TCP pores. In addition, 500TCP showed a clustering of iNOS-positive cells and CD163-positive cells at 2 weeks, suggesting the involvement of M2 macrophages in the formation of bone tissue in the TCP pores. In conclusion, we demonstrated for the first time that the geometrical structure of the artificial biomaterial, i.e., the pore size of honeycomb TCP, affects the polarization of M1/2 macrophages and bone tissue formation in TCP pores.

## 1. Introduction

In the fields of plastic surgery and oral surgery, it has been reported that there is no better grafting material than autologous bone in terms of bone induction and bone conduction ability for bone repair and bone regeneration treatment of bone defects [1]. However, artificial biomaterials have been widely used as substitutes because autogenous bone grafting requires complex harvesting procedures and is invasive to the donor tissue [2]. Recently, many preclinical and clinical trials have been conducted along with the development of bone replacement agents, and based on the results of these trials, bone replacement agents are selected and clinically applied based on clear criteria according to the case. In fact, calcium phosphate-based synthetic materials such as hydroxyapatite (HAP) [3,4], tricalcium phosphate (TCP) [5], and biphasic calcium phosphate (BCP) [6,7] are already in clinical application.

In recent years, several studies have focused on the geometrical structure of biomaterials, because in addition to the composition, an optimal geometrical structure is considered important for inducing cell differentiation and proliferation [8,9,10,11,12,13]. In view of this, we have already succeeded in developing a new biomaterial, honeycomb TCP, containing through-holes of various diameters. In our previous study, we reported that the surface properties resulting from different sintering temperatures affected the osteoinductivity and biocompatibility of TCP [14]. Furthermore, changing the through-holes diameters of honeycomb TCP holes allows for control of the cartilage and bone formation [15]. This study’s results showed that honeycomb TCP with 300 and 500 μm pore sizes and a high content of BMP-2 (1000 ng) induced bone tissue formation, while honeycomb TCP with 75 μm pore size and a low content of BMP-2 (125 ng) induced cartilage tissue formation [16]. In particular, in the rat zygomatic bone defect model, remarkable bone tissue formation was observed in honeycomb TCP containing through-and-through holes with diameters of 300 μm, suggesting its clinical applicability [17]. These findings indicate that this honeycomb TCP can potentially act as a bioactive carrier and reproduce the interactions between progenitor cells and the extracellular matrix microenvironment.

Recently, it has been reported that the polarization balance of M1/M2 macrophages with different functions is important in osteogenesis and bone repair processes [18]. Macrophages are not only responsible for innate immune responses but are also essential for repair after muscle injury and the maintenance of tissue homeostasis. Macrophages are broadly classified into pro-inflammatory M1 macrophages and tissue repairing M2 macrophages, which play important roles in the processes of inflammation, immunity, and tissue repair, respectively. M1 macrophages have been shown to produce high levels of inflammatory cytokines, have the ability to mediate resistance to pathogens, and have strong bactericidal properties, high production of active nitrogen and oxygen intermediates, and to promote Th1 responses [19,20]. On the other hand, M2 macrophages are characterized by their involvement in parasite infection defense, tissue remodeling, immunomodulation, tumor promotion, and efficient phagocytic activity [19]. Thus, it has been reported that M1/M2 macrophages, which are responsible for bone immunity during osteogenesis and fracture healing processes, have antagonistic functions, and that the M1/M2 balance is important in the microenvironment during fracture healing [21]. However, the details of the influence of macrophage polarization on bone formation using engineered biomaterials, especially with respect to the geometric structure of the artificial biomaterials, are unknown.

In this study, we investigated the role of macrophages in bone tissue using an artificial biomaterial, honeycomb TCP, and the effect of the geometric structure of the artificial biomaterial on the interaction between bone formation and macrophages.

## 2. Materials and Methods

### 2.1. Preparation of Honeycomb TCP

In this study, we used honeycomb TCP containing 300 and 500 μm pore diameters, which showed high osteoinductivity in our previous studies [16].

**Figure 1:**
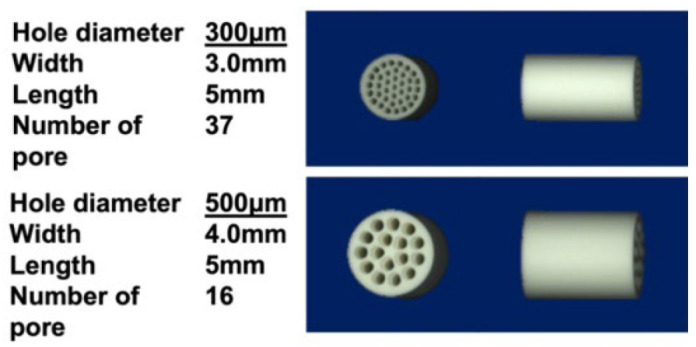
The honeycomb TCP structures used in these experiments.

Honeycomb TCP used in ectopic experiments was pressed in a cylindrical mold containing through-and-through holes with diameters of 300 μm (300 TCP) and 500 μm (500 TCP) (Figure 1). Each TCP was calcinated by sintering to 1200 °C. Details of the TCP production have been described previously [14].

### 2.2. Animals and Implantation Procedure

A total of 14 4-week-old healthy male Wister rats were used in this experiment. The 300 TCP and 500 TCP were transplanted into rat femoral muscles. The animals were randomly divided into 300TCP and 500TCP groups. The TCPs used in these experiments were loaded with BMP-2 diluted to a final contained amount of 1000 ng in AteloCell^®^ (KOKEN, Tokyo, Japan).

All of the experiments were performed in accordance with Okayama University’s Policy on the Care and Use of Laboratory Animals and approved by the Animal Care and Use Committee. All surgical procedures were performed under general anesthesia in a pain-free state.

### 2.3. Histological Procedure

For the histological observations, the implanted TCPs were removed after 1 week or 2 weeks and fixed in neutral buffered formalin. Next, the specimens were decalcified in 10% ethylenediaminetetraacetic acid for 3 weeks and then embedded in paraffin. Finally, the sections were stained with hematoxylin–eosin (HE) and observed histologically.

### 2.4. Immunohistochemical (IHC) Staining

Immunohistochemistry (IHC) staining for IBA-1 (M0 macrophage marker) [22,23], iNOS (M1 macrophage marker) [24,25], and CD163 (M2 macrophage marker) [26,27] was carried out as follows. The sections were deparaffinized in a series of xylene for 15 min and rehydrated in graded ethanol solutions. Endogenous peroxidase activity was blocked by incubating the sections in 0.3% H_2_O_2_ in methanol for 30 min. Antigen retrieval was achieved by heating in 0.01 mol/L citrate buffer for 1 min. Sections were incubated with primary antibodies as follows: rabbit anti-IBA-1 (1:100; Proteintech, Rosemont, IL, USA), rabbit anti-iNOS (1:200; Proteintech, Rosemont, IL, USA), and rabbit anti-CD163 (1:200; Abcam, Waltham, MA, USA) overnight at 4 °C. Tagging of primary antibody was achieved by the subsequent application of antirabbit IgG (ABC kit; Vector Laboratories, Inc., Newark, CA, USA). Immunoreactivity was visualized using diaminobenzidine (DAB)/H_2_O_2_ solution (Histofine DAB substrate; Nichirei Biosciences, Inc., Tokyo, Japan), and the slides were counterstained with Mayer’s hematoxylin.

## 3. Results

### 3.1. Comparison of HE Staining between 300 TCP and 500 TCP

**Figure 2:**
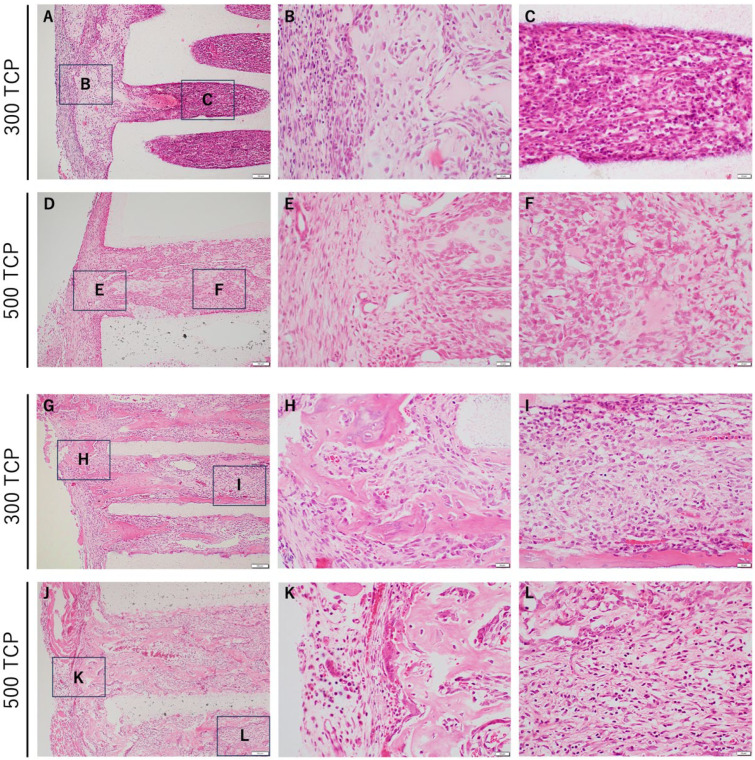
Histological images of formed tissue in TCP. (**A**) Low magnification image of 300 TCP at 1 week. Bone tissue formation was observed in the entrance of the 300 TCP pores, and granulation tissue was observed in the 300 TCP pores. (**B**,**C**) Higher-magnification images of corresponding outlined areas in (**A**). (**D**) Low magnification image of 500 TCP at 1 week. (**E**,**F**) Higher magnification images of the corresponding outlined areas in (**D**). (**G**) Low magnification image of 300 TCP at 2 weeks. Bone tissue formation was observed in the entrance of the 300 TCP pores, and bone tissue was observed in the 300 TCP pores. (**H**,**I**) Higher-magnification images of corresponding outlined areas in (**G**). (**J**) Low magnification image of 500 TCP at 2 weeks. (**K**,**L**) Higher magnification images of the corresponding outlined areas in (**D**).

In 300 TCP, cartilage and bone formation were observed near the entrance of the pores at 1 week. At 2 weeks, osteogenesis was observed near the TCP entrance, bone formation was observed within the TCP pore as if added to the TCP wall, and capillary-rich tissue formation was observed in the sparse connective tissue in the central area (Figure 2A–C).

In 500 TCP, hard tissue formation, mainly cartilage, was observed near the entrance of the pores at 1 week. At 2 weeks, bone formation was observed from near the TCP entrance to the center of the pore, adding to the TCP wall and filling the TCP pore (Figure 2D–F).

### 3.2. Immunohistochemistry Analysis of M0 Macrophage Induced by 300 TCP and 500 TCP

**Figure 3:**
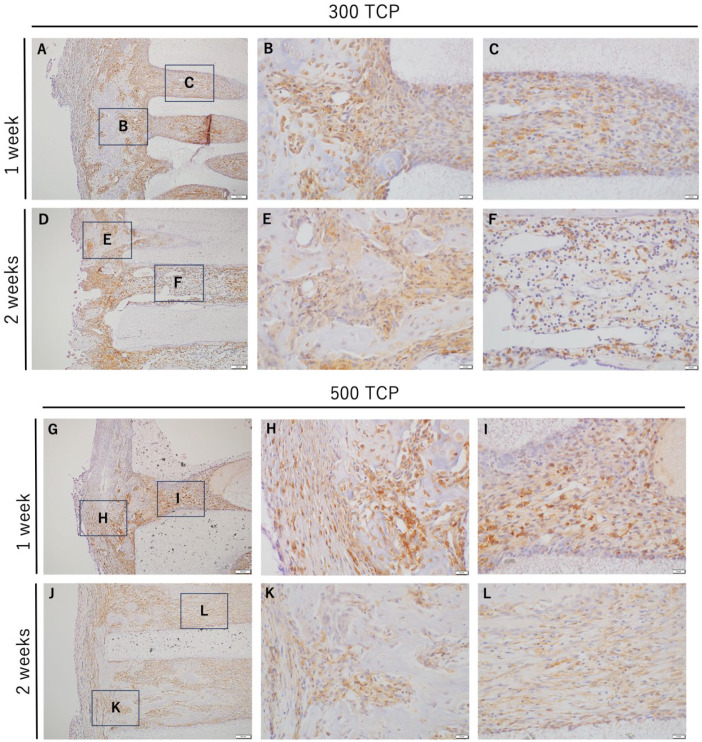
IHC images of IBA-1 staining. (**A**,**D**) Low-magnification images of 300 TCP at 1 or 2 weeks. (**B**,**C**) Higher magnification images of corresponding outlined areas in (**A**). (**E**,**F**) Higher magnification images of corresponding outlined areas in (**D**). (**G**,**J**) Low magnification images of 500 TCP at 1 or 2 weeks. (**H**,**I**) Higher magnification images of corresponding outlined areas in (**G**). (**K**,**L**) Higher magnification images of corresponding outlined areas in (**J**).

In both 300 TCP and 500 TCP, marked infiltration of IBA-1 positive cells was observed in the granulation tissue formed from the entrance of the TCP pore to the inside of the pore at 1 week (Figure 3A–C,G–I). At 2 weeks, many IBA-1-positive cells were observed around the TCP entrance in the 300 TCP and 500 TCP, as at 1 week.

However, at 2 weeks in 300 TCP, in the vascular-rich sparse connective tissue formed within the TCP pores, the number of IBA-1-positive cells decreased compared to at 1 week (Figure 3D–F). And at 2 weeks in 500 TCP, within the TCP pore, the number of IBA-1-positive cells decreased in the fibrous connective tissue compared to at 1 week, although they were observed mainly around the hard tissue formed in the pore (Figure 3J–L). The number of IBA-1-positive cells was similar between 300 TCP and 500 TCP at 1 week; however, more IBA-1-positive cells were observed in 300 TCP than in 500 TCP at 2 weeks.

### 3.3. Immunohistochemistry Analysis of M1 Macrophages Induced by 300 TCP and 500 TCP

**Figure 4:**
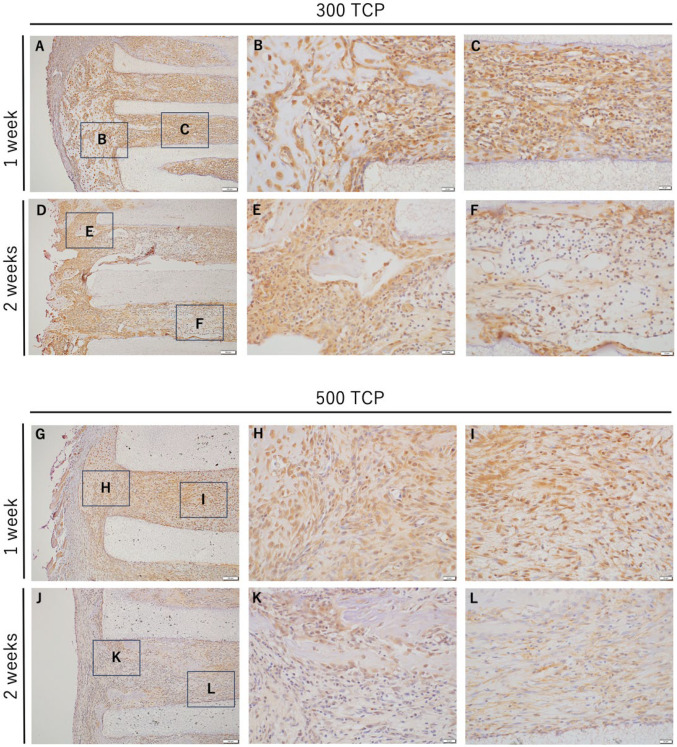
IHC images of iNOS staining. (**A**,**D**) Low-magnification images of 300 TCP at 1 or 2 weeks. (**B**,**C**) Higher magnification images of corresponding outlined areas in (**A**). (**E**,**F**) Higher magnification images of corresponding outlined areas in (**D**). (**G**,**J**) Low magnification images of 500 TCP at 1 or 2 weeks. (**H**,**I**) Higher magnification images of corresponding outlined areas in (**G**). (**K**,**L**) Higher magnification images of corresponding outlined areas in (**J**).

In both 300 TCP and 500 TCP, at 1 week, marked infiltration of iNOS-positive cells was observed in the granulation tissue formed from the entrance to the pore of the TCP. iNOS-positive cells were markedly observed around the hard tissue formed at the entrance of the TCP. Similarly, a large number of iNOS-positive cells were observed in the vicinity of the TCP entrance in 300 TCP. However, at 2 weeks, in the vascular-rich sparse connective tissue formed within the TCP pores, the number of iNOS-positive cells decreased compared to at 1 week (Figure 4).

At 2 weeks in 500 TCP, a large number of iNOS-positive cells were observed at the pore entrance as at 1 week; however, within the TCP pore, mainly around the hard tissue formed, the number of iNOS-positive cells decreased in the fibrous connective tissue compared to at 2 weeks (Figure 4G–L).

### 3.4. Immunohistochemistry Analysis of M2 Macrophages Induced by 300 TCP and 500 TCP

**Figure 5:**
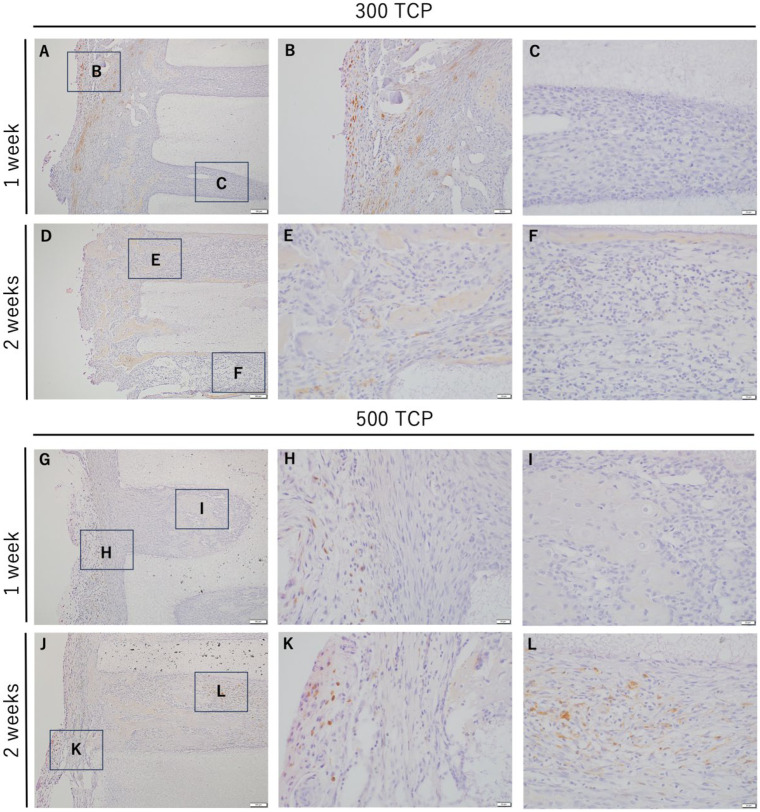
IHC images of CD163 staining. (**A**,**D**) Low magnification images of 300 TCP at 1 and 2 weeks. (**B**,**C**) Higher magnification images of corresponding outlined areas in (**A**). (**E**,**F**) Higher magnification images of corresponding outlined areas in (**D**). (**G**,**J**) Low magnification images of 500 TCP at 1 and 2 weeks. (**H**,**I**) Higher magnification images of corresponding outlined areas in (**G**). (**K**,**L**) Higher magnification images of corresponding outlined areas in (**J**).

CD163-positive cells were observed in the granulation tissue around TCP; however, CD163-positive cells were not observed at the entrance of TCP or in the TCP pores at 1 week for both 300 TCP and 500 TCP. And no CD163-positive cells were observed at the TCP inlet or in the pores at 2 weeks for 300 TCP, as at 1 week (Figure 5).

On the other hand, 500 TCP showed only a few CD163-positive cells in the immature fibrous connective tissue, which did not form hard tissue, within the pores at 2 weeks (Figure 5J–L).

## 4. Discussion

In this study, 300 TCP showed a marked infiltration of iNOS-positive cells, which are thought to be M1 macrophages, during the osteogenesis process, while no involvement of CD163-positive cells, which are thought to be M2 macrophages, was observed in the TCP pores. In addition, 500 TCP showed a clustering of iNOS-positive cells and CD163-positive cells at 2 weeks, suggesting the involvement of M2 macrophages in the formation of bone tissue in the TCP pores. In conclusion, we demonstrated for the first time that the geometrical structure of the artificial biomaterial, i.e., the pore size of honeycomb TCP, affects the polarization of M1/2 macrophages and bone tissue formation in the TCP pores.

Macrophages are involved in the body’s acute response to all biomaterials, i.e., recognition, lysis, and phagocytosis of materials, and contribute to inflammatory and tissue response processes, as well as inflammatory control and repair processes [28]. This role is largely due to the balance between the polarization of the inflammatory M1 and anti-inflammatory M2 subtypes. M1 macrophages secrete many inflammatory cytokines and promote inflammatory changes, while M2 macrophages suppress inflammatory changes by producing the anti-inflammatory cytokine IL-10 and Arg, which inhibits NO biosynthesis, and are involved in tissue regeneration [29,30]. Recently, it has become clear that the localization and the quantitative and morphological changes of osteomacrophages (OsteoMacs) are also involved in osteoblast differentiation and bone modeling and remodeling during bone repair and bone regeneration [29]. The polarity of macrophages, which are involved in the artificial bone material, is also being investigated to see if controlling the polarity of macrophages may promote bone regeneration [29]. Chen et al. reported that BCP may contribute to bone formation with a CD206- and Arg-positive so called M2-type tendency, while β-TCP showed CC chemokine receptor 7 (CCR7, CD197) and inducible nitric oxide synthase (iNOS)-positive so called M1-type tendency, in in vitro and in vivo experiments [31]. Jia et al. showed that TCP have been found to promote a favorable osteoimmunomodulatory response that can shift macrophage polarization toward the M2 phenotype. In that study, their group discovered that a histone methyltransferase enhancer of zeste 1 (EZH1) was drastically downregulated in Thp1 cells stimulated by TCP, indicating that EZH1 may participate in the macrophage phenotype shifting [32]. The infiltration of CD163-positive cells was observed outside the TCP pores at 1 week and 2 weeks for both 300 TCP and 500 TCP. However, within the TCP pores, CD163-positive cells were only observed in the juvenile fibrous connective tissue of 500 TCP at 2 weeks. Our study results showed that both 300 TCP and 500 TCP showed M1-type tendency reaction because iNOS-positive cells were clearly observed in the TCP pores, but in the 500 TCP at 2 weeks, CD163-positive cells infiltrated the TCP pores and showed M2-type tendency reactions. In general, it has been reported that M2 macrophages are involved in bone tissue regeneration using engineered biomaterials; however, bone tissue regeneration using honeycomb TCP suggests a greater involvement of M1 macrophages than M2 macrophages.

The pore size of artificial scaffolds has a crucial effect in bone formation in vitro and in vivo [33,34]. Scaffolds with an average pore size of 200–350 μm have been considered optimal for bone tissue engineering [35,36,37]. Apart from their relevance for the fate of osteoprogenitor cells during the process of bone remodelling, the key regulatory roles of the pore size of bone scaffold in the host immune response have been proven [38,39]. It appears that as the pore size of the scaffold materials increases, the foreign body reaction caused by the polymer is reduced and the tissue integration is improved [40]. Garg et al. showed that the mechanism of regulating pore size in the foreign body response may be related to macrophage polarity [41]. These results indicate that the pore size of the scaffold is a more important factor controlling the polarization of mouse bone marrow-derived macrophages than the fiber diameter. Increasing the pore size of the scaffolds promoted the expression of M2 markers and decreased the expression of M1 markers [41,42]. In addition, the oxygen supply after scaffold implantation is also affected by the pore size of the scaffold. Inadequate oxygen supply will lead to chronic inflammation and affect angiogenesis, thus affecting bone remodeling [42,43]. Therefore, by selecting the appropriate pore size of the scaffold, the formation of a hypoxic microenvironment can be avoided and the angiogenic effect can be maintained. Our previous study results suggested that the smaller vascular area induced in the pores of 300 TCP compared to 500 TCP resulted in less oxygen being carried by the vessels, resulting in hypoxia in the pores of 300 TCP and thus reproducing optimal conditions for bone marrow tissue. It was thought to mimic a hypoxic environment similar to that within the jawbone [44]. Therefore, it was considered that 300 TCP showed bone tissue regeneration mainly through infiltration of M1 macrophages, while 500 TCP showed a normal type of bone tissue formation with infiltration of M2 macrophages.

And macrophages have been reported to renew plaque calcification by enhancing osteogenic signals secreted by vascular smooth muscle cells, and macrophages involved in bone formation have been reported previously [45].

Generally, iNOS has been reported as a marker for M1 macrophages, but it has also been reported as a marker for M2d macrophages. Recently, four subtype classifications of M2 macrophages have been established: M2a, M2b, M2c and M2d, which differ from each other based on cell surface markers, secreted cytokines, and biological functions [46]. M2a is involved in cell proliferation and tissue repair, M2b acts to regulate the immune response and inflammation, and M2c promotes fibrosis renewal through TGF-β and phagocytosis of apoptotic cells. M2d has been reported to promote IL-10 and VEGF secretion and to have a pro-angiogenic effect on angiogenesis and tumor progression [46]. M2d macrophages, which are induced by the adenosine A2A receptor and TLR2, 4, 7 and 9-mediated stimulation, have been reported. M2d macrophages are distinguished from other M2 macrophages by their strong expression of vascular endothelial growth factor (VEGF), IL-10 and inducible nitric oxide synthase (iNOS) [47,48]. Furthermore, M2a and M2c macrophages have been reported to be positive for CD163. iNOS-positive cells are also considered to be markers of M1 macrophages as well as M2d macrophages. In the results of this study, 300 TCP induced macrophage polarization involved in the coupling of angiogenesis and osteogenesis, whereas 500 TCP induced macrophage polarization related to bone tissue formation, mimicking the tissue repair process, and thus promoting bone tissue formation.

## Data Availability

The data are available in a publicly accessible repository.
